# Are we nearly there yet? Starts and stops on the road to use of polygenic scores

**DOI:** 10.1007/s12687-023-00672-w

**Published:** 2023-09-27

**Authors:** Sowmiya Moorthie, Daphne Oluwasen Martschenko, Segun Fatumo

**Affiliations:** 1 PHG Foundation, University of Cambridge, 2 Worts Causeway, Cambridge, CB1 8RN UK; 2 Cambridge Public Health, University of Cambridge School of Clinical Medicine, Forvie Site, Cambridge Biomedical Campus, Cambridge, United Kingdom CB2 0SR; 3 Department of Pediatrics, Stanford Medicine, Center for Biomedical Ethics, Stanford University, Stanford, CA USA; 4 The African Computational Genomic (TACG) Research Group, MRC/UVRI and LSHTM Uganda Research Unit, Entebbe, Uganda; 5 Department of Non-Communicable Disease Epidemiology, London School of Hygiene and Tropical Medicine, London, UK

As technological advancements expand the accessibility and availability of molecular genetic data, excitement over the potential use of genetic risk scores also known as polygenic scores (PGS) or polygenic indices for disease prevention has grown. At the same time, however, the translation of PGS into healthcare and social settings raises a host of social, ethical, and clinically relevant questions. The articles in this collection examine the practical, social, and ethical implications of PGS across healthcare settings and different populations. As illustrated by the articles in this collection, uncertainty remains regarding the transferability, utility, and validity of PGS and how to responsibly adopt and implement this technology.

Genomic research is progressing rapidly as researchers search for mechanisms to integrate genomic data into healthcare and society. Genome-wide association studies (GWAS), for instance, have, and continue to lead to the identification of many common single nucleotide polymorphisms (SNPs) associated with different diseases and traits. Additionally, the growing availability of larger datasets, coupled with advances in statistical methodologies, are enabling researchers to construct polygenic score models. These are predictive models that combine information across multiple low-penetrance SNPs. The output of these models, also referred to as polygenic risk scores, polygenic indices, and genetic risk scores, provide a single measure of the cumulative effect of many individually low-impact genetic changes. Such models and their outputs have been put forward as a mechanism for assessing a person’s chances of developing a health, behavioral, or social outcome.

Nevertheless, uncertainty about the utility and validity of this tool raises questions about the value of PGS as part of healthcare. Koch et al. (2023) in their article on current prospects of PGS, discuss how despite the large number of polygenic score models developed and reported, there is limited evidence of use within clinical practice. This is unsurprising, given that PGS are the subject of divisive academic debate. For example, whilst progress has been made in developing PGS for cancer, there are still uncertainties about its use (Pashayan et al. 2023).

Much of this stems from uncertainties on how to evaluate products and tests that provide or incorporate a polygenic score (Moorthie et al. 2023). Indeed, poorly validated industry uses of PGS for prenatal embryo selection in the USA, while appealing to the American public, have concerned researchers (Johnston and Matthews 2022) and led the American College of Medical Genetics to issue a statement on their use (ACMG Board of Directors 2021). In addition, there is concern that PGS, like other genetic technologies, may exacerbate existing health inequities; Eurocentric biases continue to plague the underlying datasets used to develop polygenic score models (Martin et al. 2019). Research has shown that the less genetically similar a population is to the study sample, the weaker the predictive performance of a polygenic score model. Given this “problem of portability”, the global accessibility and generalisability of genomic advances like PGS are a major concern (Fatumo et al. 2022).

However, ensuring that such products are more widely applicable requires considering and addressing a broad range of factors. This requires a multidisciplinary approach, along with recognition and understanding of the different contexts where such products may be implemented (Cornel et al. 2014).

In many ways, PGS raise issues that are familiar to the field of human genetics (Chapman 2022). At the same time, however, there are particular concerns around the ways in which information from PGS are communicated and whether or not their use will lead to health inequities. It is important to bear in mind, that in addition to the technology itself, how programs that use PGS information are designed, and how information about this technology is communicated, can impact equity. In their paper on the role of PGS in breast cancer risk perception and decision-making, Riddle et al. (2023) reinforce this point.

The importance of engaging with communities in the design of PGS services is an increasingly important component of securing just and equitable research translation to clinic. However, as described by Wand et al. (2023) efforts to achieve meaningful engagement cannot be taken lightly; they require transparent and shared decision making, reciprocal and trusting relationships, co-learning, institutional commitment, and resources. There are many efforts underway in identifying the best mechanisms to communicate information from genomic tests across diverse communities. Mason et al. describe another effort to enhance research communication about PGS to patients and the public using theatrical performance (Mason et al. 2023). Developing a holistic understanding of the ethical and social issues that may accompany PGS implementation can also be supported by public and community engagement.

In short, it remains unclear whether PGS can provide useful information for the care of individuals and patients as part of healthcare pathways. The field will need to continue to develop the scientific research around polygenic score models and clinical applications in ways that support greater and more equitable benefit sharing. In parallel, it is also important to understand how such products will be deployed and used across different global settings. Such efforts can help put in place mechanisms to ensure products that are applicable, beneficial, and accessible to wider communities. Similar to other areas of genomic medicine, it is unlikely that there will be a one size fits all solution. Nevertheless, building on current efforts that share learning and best practice across global communities is an important part of translational research.
